# Efficient protein alignment algorithm for protein search

**DOI:** 10.1186/1471-2105-11-S1-S34

**Published:** 2010-01-18

**Authors:** Zaixin Lu, Zhiyu Zhao, Bin Fu

**Affiliations:** 1 Department of Computer Science, University of Texas-Pan American, Edinburg, TX 78539, USA; 2 Department of Computer Science, University of New Orleans, New Orleans, LA 70148, USA

## Abstract

**Background:**

Proteins show a great variety of 3D conformations, which can be used to infer their evolutionary relationship and to classify them into more general groups; therefore protein structure alignment algorithms are very helpful for protein biologists. However, an accurate alignment algorithm itself may be insufficient for effective discovering of structural relationships among tens of thousands of proteins. Due to the exponentially increasing amount of protein structural data, a fast and accurate structure alignment tool is necessary to access protein classification and protein similarity search; however, the complexity of current alignment algorithms are usually too high to make a fully alignment-based classification and search practical.

**Results:**

We have developed an efficient protein pairwise alignment algorithm and applied it to our protein search tool, which aligns a query protein structure in the pairwise manner with all protein structures in the Protein Data Bank (PDB) to output similar protein structures. The algorithm can align hundreds of pairs of protein structures in one second. Given a protein structure, the tool efficiently discovers similar structures from tens of thousands of structures stored in the PDB always in 2 minutes in a single machine and 20 seconds in our cluster of 6 machines. The algorithm has been fully implemented and is accessible online at our webserver, which is supported by a cluster of computers.

**Conclusion:**

Our algorithm can work out hundreds of pairs of protein alignments in one second. Therefore, it is very suitable for protein search. Our experimental results show that it is more accurate than other well known protein search systems in finding proteins which are structurally similar at SCOP family and superfamily levels, and its speed is also competitive with those systems. In terms of the pairwise alignment performance, it is as good as some well known alignment algorithms.

## Background

Proteins show a great variety of 3D conformations, which are necessary to support their diverse functional roles. Protein sequences and structures have close relationship with their biological functions, while protein structures reveal more evolutionary information than protein sequences do, since the structure of a protein changes more slowly in the evolution than its sequence does. Also, researchers frequently find that proteins with low sequential similarity are structurally homogenous. Therefore it is particularly important to discover the structural similarity/dissimilarity among different proteins. The research of protein 3D structure similarity provides fundamental and very helpful tools for many biological research topics, such as predicting the functions of newly discovered proteins from the functions of known similar protein structures, identifying protein families with common evolutionary origins, and understanding the variations among different classes of proteins.

Protein structures can be determined *via *experimental techniques such as X-ray crystallography, Nuclear Magnetic Resonance (NMR) spectroscopy, and even cryo-electron microscopy. Due to these techniques, the number of proteins discovered by biologists has increased dramatically over the last 30 years. The rapid growth of the PDB (see Figure 2a of [1] for an illustration of the PDB growth rate from 1970's to the year 2005) necessitates the development of efficient and accurate protein structure comparison and search algorithms and automatic software tools.

**Figure 2:**
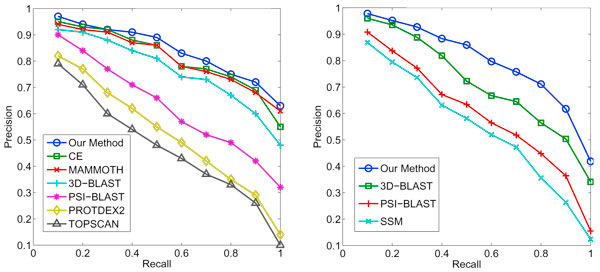
**Precision and recall curves**. Figure 2 shows the accuracy performance of multiple protein search methods. The left shows the precisions and recall rates of 108 queries by multiple methods at SCOP family level, and the right shows those of 129 queries by the same methods at SCOP superfamily level.

In order to compare the structural similarity between proteins, current protein structure alignment algorithms (e.g. [2-19]) usually try to align the *C*_*α *_atoms in protein backbones. An alignment is characterized by (1) how many atoms are matched, (2) where their positions are, and (3) how well they are matched. (1) and (2) are available once an alignment is determined. For (3), a transformation based alignment algorithm usually calculates RMSD, namely, the root mean square distance between aligned (and transformed) *C*_*α *_atoms in the structures. Although it has been studied for over 30 years, the protein structure alignment problem is far from being well resolved. New approaches and improvements to existing approaches are frequently proposed (see [20-23] for some recent works). Moreover, many questions are still under active discussions.

Protein structural similarity can be used to infer evolutionary relationship between proteins and to classify protein structures into more general groups; therefore a good protein structure alignment algorithm is very helpful for protein biologists. However, a good alignment algorithm itself may be insufficient for effective discovering of structural relationships among tens of thousands of proteins.

Protein structure query (e.g. [10,12,13,24-36]) aims to find similar structures in a protein dataset according to a given query structure. Due to the large size of protein data repositories like the PDB, protein structure query requires a very fast structure alignment tool; however, the complexity of current alignment algorithms are usually too high to make a fully alignment-based search practical. For some proteins, it may take hours to days for protein structure search engines like CE [12] and DALI [37] to return a search result; a fast and accurate protein structure query tool which enables real-time structure searching in a large dataset is still in need. To improve the search speed, in recent years many methods have been designed to reduce the query time. Baker and Dauter (2004) developed SSM [8] which uses Secondary Structure Match for the pairwise structure comparison. In addition, various linear encoding methods have been applied to protein search systems. For instance, 3D-BLAST [33] developed by Yang and Tung (2006), can improve the comparison speed thousands of times as the speed of CE and DALI. Similar methods include ProtDex2 [35], Sarst [31], and TopScan [36]. These methods improve the time performance greatly. However, when being compared with pairwise alignment methods, they have weakness in accuracy. In [34] we have developed a protein structure query algorithm and tool to find similar protein structures in the PDB for any given structure. With a combination of geometric filter and 3D structure alignment, given a query protein, the algorithm can find proteins whose structures are overall similar with the query structure in the PDB in a few minutes. The geometric filter can exclude dissimilar proteins efficiently, and reduce a lot the number of times of pairwise alignments. On the negative side, it misses some significant proteins whose structures are partially similar with the given protein.

To further improve the speed and accuracy of our protein structure query tool, in this paper we propose a very fast protein structure alignment algorithm, which is suitable to do pairwise 3D alignment with all protein structure representatives in the PDB. It can find similar proteins in the PDB (of more than 130,000 protein chains) in a short time, and avoid missing similar structures. In our experiments, some exciting results have been observed when comparing our query tool with other well known protein search engines. The experimental results show that our tool is more accurate than other systems such as CE [12], Dali [37], and SSM [8] in finding proteins that are structurally similar to the query protein, and its speed is also competitive with them.

## Results and discussion

In this section, we show the experimental results for an implementation of our algorithm and its comparisons with other well known similar systems which are accessible online. The quality evaluation of protein alignment algorithm is based on its alignment length and RMSD value. The quality evaluation of protein search tool is according to its precision, recall, and query speed.

### Evaluation of the alignment results

There is no general standard for analyzing and comparing the results of different alignment algorithms, because each method uses different alignment measures. Besides the two basic measures: alignment length and RMSD value, some methods also calculate a native score for their alignment results. For instances, CE and Dali use different kinds of Z-scores as their native score. SSM has the

where *N*_*mat *_is the number of matched pairs of *C*_*α *_atoms, *N*_1 _is the number of *C*_*α *_atoms in the first protein, and *N*_2 _is the number of *C*_*α *_atoms in the second protein. The Q-score considers both alignment length and RMSD value when measuring the alignment results. Subbiah proposed a geometric match measure,

in [38]. As shown by its definition, *SAS*_*k *_also considers both alignment length and RMSD value. Lower *SAS*_*k *_means better alignment result and *k *is the degree to which the score favors. A smaller *k *can be used when longer alignment length is preferred and a bigger *k *is for smaller RMSD value. Here we have collected 224 alignment cases and used Q-score and *SAS*_*k *_to test the performance of our algorithm. The test cases were originally proposed by various papers for various testing purposes. A list file available on our website shows all the 224 cases. They include No. 1 - No. 20 (see Table III in [12]), No. 21 - No. 88 (see Table I in [24]), No. 89 (see Tables I and II in [12]), No. 90 - No. 92 (supplement to Table III in [12]), No. 93 (see Figure 5 in [12]), No. 94 - No. 101 (see Table IV in [12]), No. 102 - No. 111 (see Table V in [12]), No. 112 - No. 120 (supplement to Table V in [12]), No. 121 - No. 124 (see Table VII in [12]), No. 125 - No. 143 (see Table 1 in [11]), No. 144 - No. 183 (see Table 1 in [17]) and No. 184 - No. 224 (see Table 2 in [17]). We compare our alignment results with Dali, CE and SSM. In each test case, different alignment algorithms have different results. CE and Dali always get more aligned pairs than those of our algorithm and SSM, but their accuracy is relatively lower (having larger RMSD value). So using merely aligned pairs or RMSD value as the criterion to measure the performance of alignment algorithm makes no sense. Therefore, we calculate Q-score and *SAS*_*k *_for the alignment results of all the methods and compare the alignment results in terms of *Q*-score Difference and Average SAS_*k*_. The *Q-score Difference *is calculated by (*Q*_*score_Ours *_- *Q*_*score_X*_) where *X *is Dali, CE or SSM. And we use *k *= 1, 2 and 3 for the Average SAS_*k *_to analyze the quality of all the four methods. It should be mentioned that our method can output sequential alignments and non-sequential alignments, thus we compare both of them with other methods. We call the sequential method SPSA and the non-sequential method NPSA for short.

**Table 1:** Results of multiple alignment algorithms. Comparison of the average alignment length, RMSD and *SAS*_*k*_.

	Dali	CE	SSM	SPSA	NPSA
Average alignment length	130.43	132.82	117.78	119.20	122.65
Average RMSD	2.78	2.83	2.37	2.23	2.30

Average SAS_1_	2.96	3.08	2.76	2.62	2.48
Average SAS_2_	3.89	3.60	3.92	3.43	3.25
Average SAS_3_	6.67	5.69	6.84	5.60	5.19

**Table 2:** Statistics on the reliability of scores. Precision is defined as *n/N *and recall rate is defined as *n/T*, where *n *is the number of true proteins of Q-scores higher than the limit value in the result list. A true protein means it is from the same family or superfamily of the query protein. *N *is the total number of retrieved proteins whose Q-scores are higher than the corresponding value, and *T *is the total number of proteins in the family or superfamily of the input protein.

Q-Score	0.9	0.8	0.7	0.6	0.5	0.4	0.3	0.2
avg.recall(%)-family	14.65	20.20	28.14	39.28	48.99	62.05	75.84	84.31
avg.precision(%)-family	99.35	98.56	97.42	97.16	94.83	91.57	87.43	70.85
avg.recall(%)-superfamily	9.22	12.89	18.96	27.53	34.87	45.22	56.59	68.43
avg.precision(%)-superfamily	99.39	99.15	99.01	98.89	98.74	97.58	96.60	87.39

### Discussion on the alignment results

**Figure 1:**
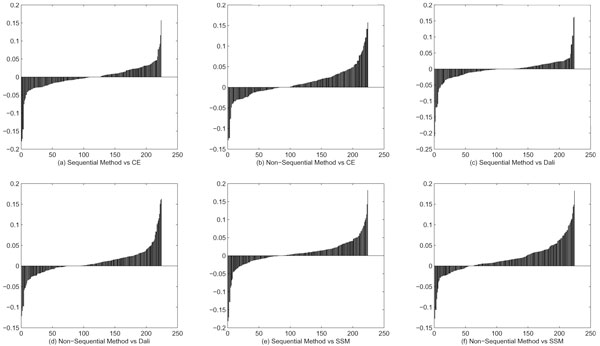
**Q-score difference plots**. Figure 1 shows the *Q*-score Difference between our algorithm and CE [12], Dali [37], and SSM [8], respectively.

Table 1 shows some statistical data based on the experimental results. Compared with Dali, CE and SSM, our algorithm has smaller average RMSD, and its average alignment length is longer than that of SSM, but shorter than that of CE and Dali. Its average SAS_*k *_is always smaller than other three algorithms, no matter *k *= 1, 2 or 3. A lower SAS_*k *_score indicates a better alignment. To further test our algorithm, we compare it with others by Q-score. Figure 1 reflects the *Q*-score Difference between our algorithm and others respectively. A black area below X-axis indicates that the Q-score of our algorithm is lower than that of the compared method. Since in each graph the upper part of the whole black area is always larger than or equal to the lower part, it is clear that our alignment algorithm is comparable to other well known algorithms. It is worth mentioning that in most test cases, SSM and our method always give alignment results with small RMSD value and shorter alignment length, while CE and Dali always find more matched pairs but with larger RMSD value. So if more matched pairs are desirable, Dali and CE are good alignment tools; on the other hand, if shorter but more accurate alignments are preferred, SSM and our method are better.

### Evaluation of the query accuracy

When applying alignment algorithms into protein search systems, the algorithms which can output best alignment results might not have the best performance of search accuracy.

The Structural Classification of Protein database (SCOP [39-42]), manually constructed by human experts, is believed to contain accurate structural classifications. The SCOP hierarchy has the following levels: domain, family, superfamily, fold, and class.

For a database search tool, the recall rate and precision are two commonly used parameters for assessing its query quality. Precision is defined as *n*/*N *and recall rate is *n*/*T*, where *n *is the number of true proteins (from the same family of the query protein) in the result list, *N *is the total number of proteins in the result list, and *T *is the total number of proteins in the same family of the query protein in the database. Therefore, the precision is between 0 and 1, and the quality of a ranked output list is directly based on it. The recall rate is also between 0 and 1, and the missing problem of a search engine is in relation to it. In 2004, Aung and Tan [35] collected 34,055 proteins form the ASTRAL SCOP 1.59 to form a large target database, from which 108 proteins were selected as the query proteins. These query proteins are from four main classes (All-*α*, All-*β*, *α*/*β *and *α *+ *β*) of ASTRAL SCOP 1.59 and their average family size is around 80. We use these different categories of proteins to do the queries on our search engine, use Q-score as the criterion to rank the output proteins, and compare the result with that of CE [12], MAMMOTH [11], 3D-BLAST [33], PSI-BLAST [29], ProtDex2 [35], and TopScan [36]. Results for all the methods except ours are taken from [33]. Since our sequential method is much faster than its non-sequential counterpart, we just use the sequential method in our experiments. In our search system we do support both sequential and non-sequential methods.

According to the 108 query results, our method based on pairwise alignment algorithm shows better performance than others. CE and MAMMOTH are the second and third accurate methods. 3D-Blast, which is based on a linear encoding method, ranks fourth and its precision is about 5% lower than ours on average. PSI-BLAST is a classical sequence search algorithm, and its precision outperforms both TopScan and ProtDex2. However, when being compared with alignment algorithms such as CE, MAMMOTH and ours, its precision is much lower. Figure 2 (left) shows the Receiver Operating Characteristic (ROC) curves for all the methods. When the recall rate is 100%, the average precision of our method is 63%, which is the highest. In addition, all the pairwise alignment methods including CE, MAMMOTH and ours have higher precision than that of other methods, and with the increasing of recall, this trend becomes more obvious.

### Performance for searching weak similarities

In the SCOP database, proteins in a same species or domain are the most similar proteins, and then are the proteins belonging to a same family. Experts also classify weakly similar proteins into a same superfamily. Therefore, to precisely assess the efficiency of searching methods challenged by searching weakly similar proteins, we use the entire ASTRAL SCOP 1.73 as the target database, and select 129 query proteins belonging to four major classes. The average superfamily size of these 129 query proteins is around 300. We use these proteins to do queries on our search engine and also on 3D-BLAST [33], PSI-BLAST [29], and SSM [8]. We are aware that ProtDex2 [35], Sarst [31], and TopScan [36] are also famous protein search systems, however they have not updated their database for a long time. Figure 2 (right) shows the experimental results of the four methods for searching similar proteins at superfamily level, according to which our method is the most accurate one. 3D-BLAST, the second most accurate method in this experiment, has its precisions about 8.1% lower than ours on average. We claim that pairwise alignment algorithms have more advantages in finding remote homologous proteins than linear encoding method or sequential search method does. Nevertheless, according to the experimental result, our method occasionally has problem detecting related proteins in the same superfamily. About 17% query results of our search engine have serious missing problems (precision lower than 50% when recall rate is 100%), while 3D-BLAST is 35% and other methods have more serious missing problems than 3D-BLAST.

### Evaluation of reliability

Our search engine provides an alignment length and an RMSD value for every retrieved structure. In order to assess the reliability of our search engine, we calculate the Q-score value of each structure by its alignment length and RMSD value; and gather statistics on precision and recall rate for various Q-score values at both superfamily and family levels. According to the data in Table 2, when Q-score is higher than 0.4, the average precisions are over 90% for both levels. The recall rates are 62.05% for family level and 45.22% for superfamily level.

### Evaluation of query speed

As shown in Table 3, on average, our method requirs about 112.20 seconds to search the database for each query protein in a single machine. Although it is much slower than 3D-BLAST and PSI-BLAST, when being compared with pairwise alignment algorithms CE, and MAMMOTH, ours has great advantage in the time performance. In addition, we have used our web server and the web servers of 3D-BLAST, PSI-BLAST and SSM to do the 129 queries in the entire PDB database. The PSI-BLAST, SSM, and 3D-BLAST servers have average query time of 16, 27, and 44 seconds respectively. Benefit from the distributed computing system and the offline classification, our web tool can scan the entire PDB database in 17 seconds on average, which is shorter than those of 3D-BLAST and SSM. Moreover, our results contain an alignment length and an RMSD value for every output protein. Although PSI-BLAST and 3D-BLAST do not have these data, they are the most important measures for comparing protein structural similarities. The 3D-BLAST server is the slowest one with an average query time of 44 seconds. In our knowledge, other search engines such as CE [12] and Dali [37] which are based on one-against-all pairwise alignment algorithms need hours to days to complete the queries.

**Table 3:** Average search time of each program on 108 queries in the SCOP 1.59. Results for all the methods except ours are taken from [33]. Their experiments were performed on a computer with an Intel Pentium 2.8 GHz processor and 1,024 megabytes of RAM memory. Ours were done on a computer with Intel Pentium 2.66 GHz processor and 1,024 megabytes of RAM memory.

Software	Total search time (s)	Average search time per query (s)
Our method	12,117	112.20
3D-Blast	34.35	0.318
PSI-BLAST	18.31	0.170
CE	13.5 days	3 hours
MAMMOTH	131,855	1220.88

## Conclusion

We have developed a fast protein alignment algorithm and an efficient protein structural similarity search engine by a combination of the structure alignment algorithm and a structure classification method. Our experiments show that it is more accurate than other well known systems in finding proteins that are structurally similar. The fast speed of our alignment algorithm results from a simple and efficient method for finding a rigid body transformation.

## Methods

### Brief description of algorithms

**Figure 3:**
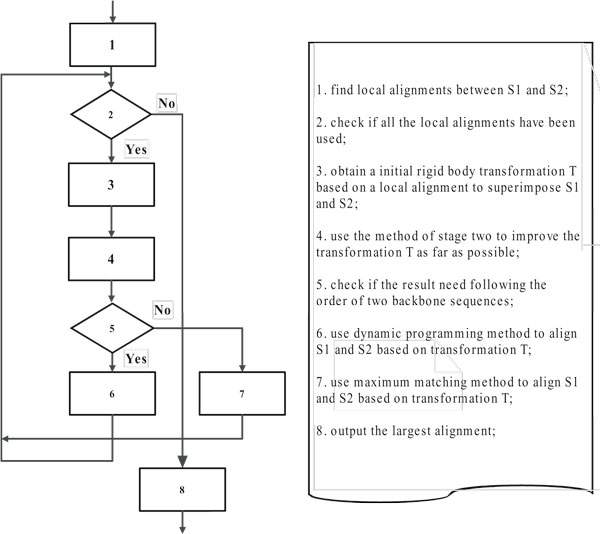
**Flowchart**. Figure 3 is a flowchart of our alignment algorithm.

We give a brief description of our algorithms in this section. We have developed two protein alignment algorithms, both of them have three main stages. The first two stages are shared by them, but they have a different third stage. The third stage of the first alignment algorithm is based on DP (dynamic programming), and it preserves the order of *C*_*α *_atoms in protein backbones. The second algorithm, whose third stage is based on MM (maximal matching), does not preserve the order, however it brings larger alignments than the first, while its speed is slightly slower. Our main technical contribution is a fast method used in stage two for finding a rigid body transformation to superimpose two protein structures. A process flow is shown in Figure 3.

### Brief-Algorithm

*S*_1 _and *S*_2 _are two 3D protein *C*_*α *_backbone structures; *L *is a set of local alignments (a local alignment is a match of two substructures of consecutive *C*_*α *_atoms from two backbones); *δ *is the maximum allowed distance between two matched *C*_*α *_atoms; *F*_*G*_() is a function to calculate rigid body transformation; *T *() is a function to translate and rotate a structure; find local alignments (stage 1);

for each local alignment *l *in *L*

   *T *= *F*_*G*_(*l*) (beginning of stage 2);

    = *T*(*S*_2_);

   repeat

      *H *= ∅;

      for every pair of points (*p*_*u*_, *q*_*v*_), where *p*_*u *_is in *S*_1 _and *q*_*v *_is in *S*_2_

         if *distance*(*p*_*u*_, *q*_*v*_) ≤ *δ *then put (*p*_*u*_, *q*_*v*_) into *H*;

      end for

      *T *= *F*_*G*_(*H*);

       = *T*(*S*_2_);

   until the number of pairs in *H *does not increase (end of stage 2)

   find aligned pairs between *S*_1 _and  from *H *(stage 3);

end for

output the largest alignment;

End of Algorithm

#### First stage

In the first stage, our algorithm searches for a set of local alignments, each consisting of a series of consecutive *C*_*α *_atom pairs in the backbones of two proteins *P *and *Q*, which are represented by their *C*_*α *_atoms in backbones *P *= *p*_1_*p*_2 _⋯  and *Q *= *q*_1_*q*_2 _⋯ . We use (*i*, *j*, *l*) to represent a local alignment, which indicates that a gapless segment *p*_*i*_*p*_*i*+1 _⋯ *p*_*i*+*l*-1 _of the first protein backbone *P *starting at *C*_*α *_atom *i *matches a gapless segment *q*_*j*_*q*_*j*+1 _⋯ *q*_*j*+*l*-1 _of second protein backbone *Q *starting at *C*_*α *_atom *j*, and both segments have *l *atoms. We compute the distance matrices of the two backbones to match their local regions. If all the corresponding distances in the two distance matrices have small difference, then a local alignment is found. Our algorithm to search all the local alignments runs in time *O*(*d*_1_*m*_1_*m*_2_), where *d*_1 _is a constant number, *m*_1 _is the number of *C*_*α *_atoms in *P *and *m*_2 _is the number of *C*_*α *_atoms in *Q*.

#### Second stage

In the second stage, each local alignment is used to find an initial rigid body transformation. There are many algorithms for finding a rigid body transformation to superimpose a set of pairs of 3D points [43]. In this paper a least square estimation method [44] is applied in our algorithm. Given a set *H *= {(*a*_1_, *b*_1_), (*a*_2_, *b*_2_),⋯,(*a*_*m*_, *b*_*m*_)} of point pairs in the 3D Euclidean space, *F*_*G*_(*H*) is a rigid body transformation *T *derived by the method in [44] to minimize the RMSD: . Let *H*_0 _be the set of all pairs (*p*_*i*+*k*_, *q*_*j*+*k*_) (*k *= 0, 1,⋯, *l *- 1) in a local alignment (*i*, *j*, *l*). The rigid body transformation *T*_0 _= *F*_*G*_(*H*_0_) is derived. After obtaining *T*_0_, we use it to superimpose the two structures and collect all the pairs (*p*_*u*_, *q*_*v*_), where *p*_*u *_is from *P *and *q*_*v*_is from *Q *and the distance between *p*_*u *_and *T*(*q*_*v*_) is bounded by a threshold, and put them into *H*_1_. A new rigid body transformation *T*_1_, which is *F*_*G*_(*H*_1_), is derived based on the new set *H*_1_. By repetitively calculating a new transformation and adding new point pairs into H1, we can improve the transformation until no more pairs can be added.

A lot of matched pairs are used during the process of getting a transformation. It is necessary to mention that our algorithm selects all the matched pairs without considering conflicts, where two pairs share a same *C*_*α *_atom. Obviously, this kind of conflicts is ndisallowed in a global alignment. However, when calculating a transformation, there is no need to consider that, and sometimes it is hard to choose the best one among the conflicting pairs. Our method of searching a rigid body transformation with the presence of conflicting pairs not only makes the second stage simple and fast, but also improves its accuracy.

#### Third stage

In the third stage, we output an alignment that is a set of aligned pairs, where each *C*_*α *_is allowed to appear in at most one pair. Two different methods, dynamic programming and maximal matching, are applied to bring the sequential alignment and non-sequential alignment, respectively.

The dynamic programming method can find an optimal solution by following the order of two backbone sequences. And the minimal length of a local alignment is set to 4. The maximal matching method returns a non-sequential alignment. Before applying the classical maximal matching algorithm to a graph of local alignments, we first delete edges in the graph so that each edge in the bipartite graph corresponds to a local alignment of length at least 4. The minimal length is used to exclude some isolated pairs, which are not biologically meaningful.

As we know, most existing protein alignment algorithms repeat finding the aligned regions between two backbones and recalculate the rigid body transformation when looking for a maximal alignment. Our algorithm does not involve the *C*_*α *_atoms alignment when determining the rigid body transformation. This is why our alignment algorithm is remarkably faster than other algorithms. It can work out hundreds of pairs of protein alignments in one second. Therefore, it is very suitable for protein search. The sequential and non-sequential methods show an interesting tradeoff between speed and the number of aligned pairs.

### Methods of speeding up the alignment and search

In order to speed up the computation of our basic algorithm, we propose some strategies that improve the time performance.

#### Improve the time performance of the alignment algorithm

Finding a good rigid body transformation between two protein structures is often time consuming. This is why most alignment algorithms are relatively slow. In order to develop an efficient protein alignment algorithm for protein search, we reduce computational time for finding the rigid body transformation while maintaining sufficient alignment quality.

First of all, the size of local alignments directly determines the time performance. A large number of local alignments will result in slow global alignments. Before finding the local alignments, we first filter out all the short *α *helices of length less than 8, because this kind of local structures are very common in proteins and may prevent the program from finding significant local alignments. Moreover, the length of a local alignment should be reasonably large to make sense, so we only consider local alignments with length greater than a threshold. In our experiments these two filters excluded about 85% unnecessary ones from all the possible local alignments.

Furthermore, each local alignment is used to calculate an initial rigid body transformation. However, it is possible that the final global alignments derived from different local alignments are the same or highly similar. Obviously, reducing these redundant local alignments can improve the time performance greatly. For each local alignment, we first apply every previously obtained rigid body transformation to it and calculate a corresponding RMSD value. A small RMSD by some transformation means that a final global alignment based on the current local alignment will be similar to that based on an old local, therefore the current one can be skipped. This technique efficiently reduces the unnecessary calculation of transformations. Supported by these effective filters, our alignment algorithm compares hundreds of pairs of proteins in one second, remarkably faster than other well known alignment algorithms.

#### An Offline classification

Our search system has an offline classification for all the protein structures in the PDB. The purpose of this classification is to improve the speed of protein structure query. It has the following steps:

1. Partition the PDB into groups. Use our pairwise alignment algorithm to check the structural similarity among the protein chains in the PDB. The Q-score, proposed in [8] is a non-linear score for measuring such similarity. It has been found that different protein structure query servers agree reasonably well on this score. Here we support that two proteins with alignment Q-score higher than 0.5 are similar. Therefore, after the partition every two proteins in the same group have a pairwise alignment Q-score of at least 0.5.

2. Select representatives. For each group, one protein chain is selected as a representative. In order to get it, we align each protein with all others in the same group and calculate the sum of Q-scores. A protein chain with the highest sum of Q-scores is selected as the representative. The classification is based on all-against-all pairwise alignment. It synchronizes itself with the PDB and all the protein chains in the PDB are partitioned into about 14,000 groups. Searching the entire PDB with 130,000 protein chains is reduced to doing so in our classified database with about 14,000 representatives. Thus, it improves the speed to a great extent.

#### Distributed computing

In order to speed up the query system by taking advantage of parallel computing, we assign the representative proteins of the over 14 K groups to multiple computers to perform pairwise structure alignments with the input protein simultaneously. Based on the experimental result of 129 queries, the average query time of our search tool is 104.04 seconds in a single machine, and 17.84 seconds in our cluster. The cluster has 6 computers and it achieves a linear gain of time performance that is close to 6.

## Competing interests

The authors declare that they have no competing interests.

## Authors' contributions

Zaixin Lu designed the current version of algorithm and the Java software implementation [45]. Zhiyu Zhao developed an earlier version of the protein alignment algorithm, which has been further improved in this paper. Bin Fu contributed to the theoretical part of algorithm design and organized this research.
